# A rehabilitation tool for functional balance using altered gravity and virtual reality

**DOI:** 10.1186/1743-0003-4-25

**Published:** 2007-07-10

**Authors:** Lars IE Oddsson, Robin Karlsson, Janusz Konrad, Serdar Ince, Steve R Williams, Erika Zemkova

**Affiliations:** 1 Sister Kenny Rehabilitation Institute, Sister Kenny Research Center (12101), 800 E. 28th St. Minneapolis, MN 55407, USA; 2 NeuroMuscular Research Center, Boston University, 19 Deerfield Street, Boston, MA, 02215, USA; 3 Department of Electrical and Computer Engineering, Boston University, 8 St Mary's Street, Boston, MA 02215, USA; 4 Department of Rehabilitation, Boston Medical Center One Boston Medical Center Place, Boston, MA 02118, USA; 5 Department of Sports Medicine, Institute of Sport Sciences, Faculty of Physical Education and Sports, Comenius University, Bratislava, Slovakia

## Abstract

**Background:**

There is a need for effective and early functional rehabilitation of patients with gait and balance problems including those with spinal cord injury, neurological diseases and recovering from hip fractures, a common consequence of falls especially in the elderly population. Gait training in these patients using partial body weight support (BWS) on a treadmill, a technique that involves unloading the subject through a harness, improves walking better than training with full weight bearing. One problem with this technique not commonly acknowledged is that the harness provides external support that essentially eliminates associated postural adjustments (APAs) required for independent gait. We have developed a device to address this issue and conducted a training study for proof of concept of efficacy.

**Methods:**

We present a tool that can enhance the concept of BWS training by allowing natural APAs to occur mediolaterally. While in a supine position in a 90 deg tilted environment built around a modified hospital bed, subjects wear a backpack frame that is freely moving on air-bearings (cf. puck on an air hockey table) and attached through a cable to a pneumatic cylinder that provides a load that can be set to emulate various G-like loads. Veridical visual input is provided through two 3-D automultiscopic displays that allow glasses free 3-D vision representing a virtual surrounding environment that may be acquired from sites chosen by the patient. Two groups of 12 healthy subjects were exposed to either strength training alone or a combination of strength and balance training in such a tilted environment over a period of four weeks.

**Results:**

Isokinetic strength measured during upright squat extension improved similarly in both groups. Measures of balance assessed in upright showed statistically significant improvements only when balance was part of the training in the tilted environment. Postural measures indicated less reliance on visual and/or increased use of somatosensory cues after training.

**Conclusion:**

Upright balance function can be improved following balance specific training performed in a supine position in an environment providing the perception of an upright position with respect to gravity. Future studies will implement this concept in patients.

## Background

Gait training using partial body weight support [1] (BWS) is a neurorehabilitation technique that is becoming increasingly popular and is being used to enhance locomotor recovery following a range of motor disorders associated with stroke, spinal cord injury, cerebral palsy and Parkinson's Disease as well as for early mobilization following total hip arthroplasty. Early mobilization following any injury or disease that leads to immobility is crucial for recovery and in the case of hip fractures, early ambulation has even been shown to be directly predictive of extended survival [2]. Initially proposed by [3] as a gait retraining strategy for patients with neurological impairment, the BWS approach was based on earlier work in the cat [4,5] indicating its feasibility in humans. Although work in this area is currently ongoing and the final word on treatment effectiveness of this method is still out, several recent studies show promising results. Improved mobility following training with BWS has been demonstrated in patients with spinal cord injury [6-8], stroke [9-11], cerebral palsy [12] and Parkinson's disease [13] as well as following total hip arthroplasty [11] and neck-of-femur fracture [14]. However, it appears that improvement in balance function following BWS training mainly occurs in patients with minimal function prior to treatment [10] suggesting that the BWS regimen is not sufficiently challenging for more functional patients. Consequently, the challenge to the balance system is either too small to stimulate improvement or is not sufficiently specific to balance function. In fact, differences seen in muscle activity between gait during BWS and full weight bearing at different velocities may reflect the decreased need for balance control and absence of associated postural adjustments (APAs) during BWS gait [3,15].

**Figure 1:**
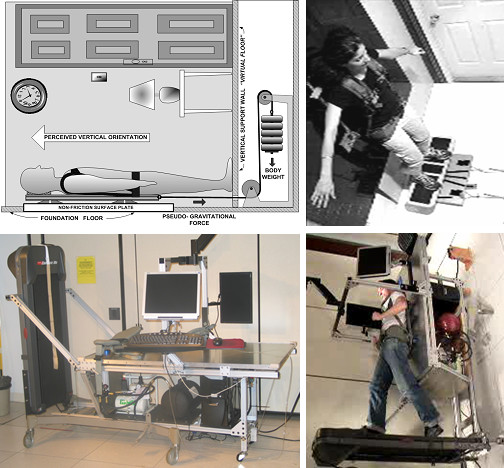
**Tilted Room environment (left) and moveable system for functional neurorehabilitation (right)**. Left: Tilted Room environment used for training study. The subject wears a back-pack frame with air-bearings allowing friction free mediolateral motion. The frame is attached to a weight stack that provides a gravity-like load that the subject must balance against. The room contains common physical objects that have a visual "polarity" with respect to gravity, i.e. we commonly align their position with gravity thereby enhancing the perception of being upright in an upright environment. Right: In the moveable bed system the subject views two large automultiscopic LCD screens that project 3-D images. The screens represent windows in a virtual room surrounding the subject. Images can be from the patient's own home, country house etc. Note that treadmill is not shown in schematic.

One issue with the BWS technique that has not been commonly recognized is that the harness supporting the subject decreases the need for natural APAs that are required for independent gait. The main site for an active control of balance during gait is the step-to-step mediolateral placement of the foot [16-18]. When supported by a harness during BWS training any mediolateral movement is restricted by a medially directed reaction force component that will help stabilize the body in the frontal plane and decrease or even eliminate the need for APAs. This restriction may limit the full advantage of unloaded gait training. To address this issue, we have designed a system that can refine the concept of BWS training by allowing natural APAs to occur spontaneously. We propose that unloaded gait training is more effective if APAs are allowed. In a pilot study we have demonstrated that upright balance function improves after training in a small (8 × 8 × 8 feet) 90 deg tilted room with the subject in a supine position strapped to a device (freely moving on air-bearings, cf. puck on an air hockey table, Figure 1, left). The room contained familiar objects providing a perception of being upright in an upright environment [19]. A G-like load was provided with a weight stack [20,21]. For movements in the frontal plane, this tilted environment requires the subject to perform APAs as if in an upright environment. No postural control is required for sagittal plane movements. A video of a subject exercising in this tilted room appears to a blinded viewer as being upright with normal frontal plane APAs to balance against gravity. Here we describe a similar system that is intended to be moveable for use in a clinical setting. Instead of a physical room, subjects view two 3-D automultiscopic displays that allow 3-D vision without any special glasses [22,23]. The screens represent windows to a virtual surrounding environment that may be acquired from sites chosen by the patient. We also present data from a training study conducted in the tilted room environment with two groups of healthy subjects. The goal of the training study was to demonstrate that training in the tilted environment can improve aspects of upright strength and balance function concurrently, a concept that could provide early functional rehabilitation for patients as well as become an effective countermeasure for training of astronauts in preparation for lunar and/or Mars missions.

## Methods

### Participants and Procedures

Two groups of healthy subjects; 1) Strength and Balance Training (S&B, 6 female+6 male, 20–21 yrs, 170.1 ± 9.2 cm, 68.6 ± 10.8 kg) and; 2) Strength Training (S, 5 female+ 6 male, 19–25 yrs, 173.5 ± 9.0 cm, 68.7 ± 10.8 kg) participated in the study. The project was approved by the Boston University Charles River Campus IRB. The S&B group performed "squats" in the tilted room environment (Figure 1, left) [21] on a balance board that required them to balance in the mediolateral direction, whereas the S group performed squats without balance requirement (sliding on fixed rails and no balance board). The strength program was progressive (50%–75% of 1RM) and each session consisted of 6 sets of 10 reps.

The following measures were conducted before and after training; 1) Maximal Voluntary Contraction (MVC) during an isokinetic squat extension (10 deg/s & 35 deg/s) using a computerized exercise system (CES, Ariel Dynamics, CA, USA); 2) Stationary stance on one leg with eyes open and with eyes closed while standing on a force platform. Ten trials of 30s standing were performed under each condition. Subjects rested between as needed between trials to minimize effects of fatigue. Subjects were instructed to stand as still as possible during each trial and to actively minimize their perceived body sway. Center of pressure (COP) data were recorded at 100 Hz. Summary statistics and Stabilogram-Diffusion parameters were extracted from the COP data [24].

## Results

### Strength and Balance Training in a Tilted Room Environment

**Figure 2:**
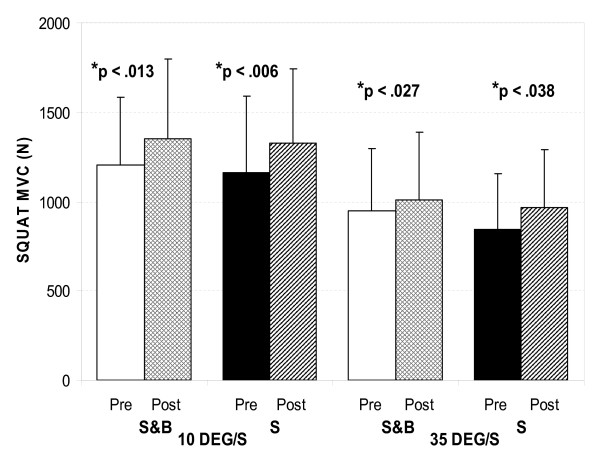
**Pre- and post-training data of MVC**. White and black graphs show pre-test MVC values for the S&B and S groups during slow (10 deg/s, left) and fast (35 deg/s, right) isokinetic squat extension, respectively. Similarly, cross-hatched and striped graphs show post test MVC values for S&B and S groups, respectively. Graphs represent mean with bars indicating+ 1 standard deviation of the mean. All pre- to post-training changes were statistically significant.

**Figure 3:**
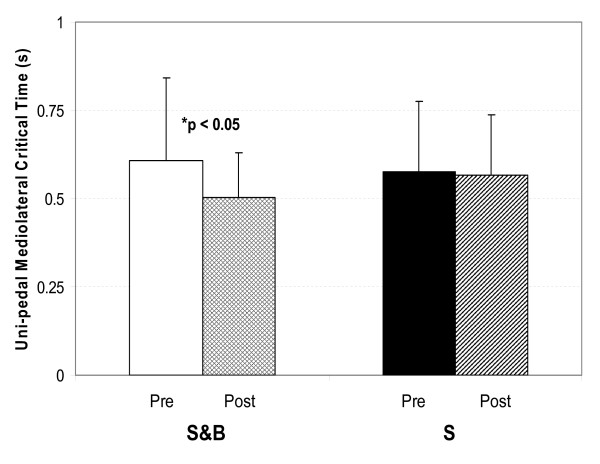
**Pre- and post-training results of the mediolateral critical time parameter**. Changes in the mediolateral critical time parameter under eyes-closed conditions are shown for the S&B group (Left) and the S group (Right). Graphs represent mean with bars indicating+ 1 standard deviation of the mean. A statistically significant change was only seen in the S&B group.

Figure 2 shows maximum isokinetic strength before and after training in the tilted environment for the two groups. Both the S&B and the S groups showed statistically significant improvements in MVC during both isokinetic velocities. Improvements at the higher velocity appeared marginally larger for the S group (Figure 2). Several subjects in the S&B group reported subjectively that they perceived improvement in their ability to control posture following the training. Measures of balance control confirmed such an improvement. Overall, effects on postural parameters were mainly seen in the mediolateral direction, specific to the direction of postural challenge in the tilted room during training. Figure 3 shows the mediolateral critical time parameter for eyes-closed conditions from the stabilogram-diffusion analysis. This parameter indicates the time interval at which, on average, the random walk behaviour of the COP changes from being predominantly persistent (tendency to continue in the same direction) to being predominantly antipersistent (tendency to reverse direction). The critical time parameter was 105 ms shorter after training for the S&B group (p < 0.05, Figure 3) with a non-significant decrease of 9 ms in the S group. A similar, although non-significant, decrease was seen with eyes open in the S&B group (p < 0.14).

The combined S&B training appeared to alter the relationship between balance performances under eyes closed vs. eyes open (Romberg ratio). The Critical Displacement parameter, indicating the average COP displacement at which the postural control process becomes mainly antipersistent, was five times higher under eyes closed compared to eyes open pre-training for the S&B group and decreased by 30% to 3.5 post-training (p < 0.04). There was a small non-significant decrease in the S group (6%). A post-training decrease in the S&B group of 21% (p < 0.03) was seen for the ratio between mediolateral short-term diffusion coefficients indicating a relatively lower short-term stochastic activity under eyes closed conditions as a result of the training. This was mainly related to a 40% increase in mediolateral short-term stochastic activity under open eyes conditions (p < 0.012). No change was observed for the S group.

## Discussion

The current project has demonstrated the feasibility of a novel moveable clinical device intended for functional rehabilitation of strength and balance in a wide range of categories of patients. The system allows natural mediolateral APAs to occur across a wide range of gravity-like loads, an important balance related stimulus that currently used BWS systems cannot provide. Consequently, the system would complement currently used technologies by providing a unique training stimulus that is of importance for independent upright stance and gait. An additional benefit of the system when compared to independent upright gait training is that loss of balance will not lead to a fall. Therefore, frail patients who are at high risk of re-injury in case of a fall during upright rehabilitation would be able to perform balance rehabilitation, independently with no assistive devices (canes, walker or support bars), no risk of a fall and minimal risk of injury. Allowing such patients to stand and/or walk independently in a safe setting may in addition to improve their physical functioning also help rebuild their confidence and therefore decrease their fear of falling and quality of life.

The results from our training study have shown, using healthy individuals, that upright strength and balance function improves concurrently following combined strength and balance training in a 90 degree tilted environment. Strength training alone did not improve balance function. It is of particular interest that balance function improved since vestibular information from the otolith organ, that normally provides tilt orientation information with respect to gravity, cannot provide any relevant such information when balancing in the tilted environment. Consequently, any balance improvement must have been related to enhanced use of somatosensory, visual and/or linear and angular acceleration information from the vestibular system. The shorter critical time parameter of the Stabilogram-Diffusion analysis seen after training in the S&B group would suggest that these enhancements allowed implementation of postural corrections that where, on average, 105 ms quicker than before training.

## Conclusion

These results support the view that combined strength and balance training in the tilted environment, where the vestibular tilt orientation mechanism cannot be used for balancing, can improve balance function during upright while balancing against gravity in addition to muscular strength. This is important to know when designing rehabilitation programs for patients who are candidates for this kind of training. In addition, this indicates that astronauts training in-flight under microgravity conditions can target postural control and may improve training efficiency by a multimodal regimen where strength training is performed under conditions where balance is challenged.

### Future Plans – Moveable Clinical System for Functional Neurorehabilitation

#### System Description

Figure 1 (lower row) shows two photographs of the recently developed system; the system on its own (left) and with a subject while walking on the permanently fastened motorized treadmill (right). Notice that the picture has been rotated 90 degrees to convey a visual effect of upright gait. A flat floor surface allowing various standing exercises can be placed over the treadmill belt. For example, a custom built balance board can be attached to the floor board through a high grade door hinge with a removable pin. The wheel base of a regular hospital bed has been modified to hold a pneumatic force actuator, a flat friction free surface that supports a back pack frame with air bearings, a visual surround system and a portable air-compressor. Subjects wear a sturdy low weight aluminum back pack frame (Aruc Systems LLC, Eau Claire, WI) mounted with three air bearings (NewWay Airbearings Aston, PA) that allow the subject friction-free movement in the frontal plane. A cable attached to the lower part of the frame runs between the legs of the subject to a pulley mounted on a linear bearing allowing mediolateral motion of the pulley before connecting to the pneumatic actuator. The bearings are porous, 2 1/2" in diameter and can support ~175 lbs each at 60 psi with 10 micron lift. Porous air bearings, typically made with carbon, provide an almost uniform air pressure across the entire bearing surface. The carbon surface also provides greater bearing protection if there is an air supply failure, and allows the bearings to be moved during air failure without damaging the support surface. The support surface consists of a 1 inch thick styrene plate sandwiched between 1/16th inch thick aluminum sheets. The pneumatic actuator can provide up to ~300 lbs of force at 50 psi air pressure. Air support can either be provided externally or from an on-board dental air compressor (Bambi Air Compressors Ltd, Birmingham, UK).

#### Three-Dimensional Display Techniques

Visual cues to convey a perception of being in an upright environment are provided through state of the art display techniques with 3-D images of a virtual environment. Typically, stereoscopic 3-D displays require polarized or shutter glasses to deliver the projected images separately to each eye. Inconvenience, often discomfort, and, in the case of shutter glasses, cost, are some of the reasons that eyewear-based 3-D displays are far from practical. Additionally, stereoscopic systems render 3-D environment from one single viewpoint thus making any viewer movement in front of the screen unnatural (static 3-D objects rotate with lateral head motion). Recently, a new type of 3-D displays, called automultiscopic, have been developed [22,23]. Such displays require no glasses and project multiple views; a viewer can clearly experience depth and even see a little around objects. These displays are capable of projecting several, typically 9, views of a 3-D scene. The current system uses two displays from Stereographics Corp (Synthagram SG222, resolution 3840 × 2400 pixels and Synthagram SG202, resolution 1600 × 1200 pixels), one placed in front of the subject and one on the side. When still images displayed on these screens they are intended to represent virtual "windows" to an outside environment and thereby promote a visually induced reorientation illusion where subjects perceive themselves as being upright with respect to gravity [19-21].

Instead of using nine cameras to generate 3-D images fewer cameras are commonly used and missing views are reconstructed by means of multi-dimensional signal processing. The nine views are then combined together in a process called "interzigging". An enhanced algorithm, incorporating two-dimensional lowpass filtering to eliminate spatial aliasing has recently been developed [25]. The fundamental issue in capturing 3-D information and then rendering it on a 3-D screen is to accurately measure the scene depth or *disparity*, the latter defined as a vector in the image plane that connects projections of the same 3-D feature in both views. Given camera parameters (baseline, focal length), depth can be computed from disparity and vice versa. The estimation of disparity is typically achieved by assuming an invariant property, such as brightness or color, and then establishing correspondence between images based on this property [26-28]. Additional work by Konrad [25,29-31] has addressed these issues. Based on disparities between two calibrated views, depth (structure) of the captured 3-D scene can be computed which, in turn, permits the reconstruction of views from virtual cameras. Reconstruction based on 2 views is a well-researched problem [32-35].

The system depicted in Figure 1 (lower row) has been built and pilot tested for functionality during gait on the attached treadmill, standing on one and two legs on the floor surface and while balancing on the balance board. G-load can be varied continuously and the Bambi compressor can provide airflow simultaneously to the air bearings and the pneumatic actuator. The system can easily be moved and handled by one person and is as wide as a regular hospital bed.

## Competing interests

Dr. Oddsson is the inventor on a provisional patent filed by Boston University on the technology presented in this manuscript. There are no other competing interests.

## Authors' contributions

LO conceived and designed the moveable bed device, designed the training study and drafted the manuscript. RK participated in the design of the moveable bed device and the tilted room, and has built the moveable bed system. JK and SI developed and designed software algorithms for displaying 3D images on the automultiscopic screens used in the moveable bed device and they helped write the manuscript. SW participated in design aspects of the moveable bed device with particular focus on patient compliance and relevance for clinical implementation. EZ participated in the design of the training study, data acquisition as well as analysis and interpretation. All authors read and approved the manuscript.
